# Tension-Free Vaginal Tape and Polyacrylamide Hydrogel Injection for Primary Stress Urinary Incontinence: 3-Year Followup from a Randomized Clinical Trial

**DOI:** 10.1097/JU.0000000000002720

**Published:** 2022-08-05

**Authors:** Anna-Maija Itkonen Freitas, Camilla Isaksson, Päivi Rahkola-Soisalo, Sari Tulokas, Maarit Mentula, Tomi S. Mikkola

**Affiliations:** 1 Helsinki University, Department of Obstetrics and Gynaecology, Helsinki University Hospital, Helsinki, Finland

**Keywords:** suburethral slings, polyacrylamide gels, surgical mesh

## Abstract

**Purpose:**

We sought to determine whether polyacrylamide hydrogel (PAHG) is noninferior to tension-free vaginal tape (TVT) in the treatment of women with primary stress urinary incontinence (SUI).

**Materials and Methods:**

In this noninferiority trial, 223 women eligible for operative SUI treatment were randomized for TVT (110) or PAHG (113). Primary outcome was patient satisfaction and the noninferiority margin for the difference was 20%. Secondary outcomes were effectiveness and complications.

**Results:**

At 3 years, 188 (84.3%) women attended the followup. The satisfaction score (visual analogue scale 0–100) median was 98.5 (IQR 90–100) in the TVT group and 90.0 (IQR 70–100) in the PAHG group, whereas a score ≥80 was reached in 87 (94.6%) and 65 (67.7%), respectively (difference 26.9%, 95% CI 16.7% to 36.8%). Thus, PAHG did not meet the noninferiority criteria set in our study. The cough stress test was negative in 88 (95.7%) of TVT patients vs 75 (78.1%) of PAHG patients (difference 17.5%, 95% CI 8.6% to 26.9%). Any peri- or postoperative complication before crossover between the groups was detected in 40 (43.5%) women in the TVT group and 23 (24.0%) women in the PAHG group (difference 19.5%, 95% CI 6.8% to 31.4%).

**Conclusions:**

In midterm followup, PAHG did not reach in patient satisfaction the noninferiority set in our study. Furthermore, mid urethral TVT slings show better subjective and objective cure rates than PAHG. However, complications were more often associated with TVT. Since the majority of PAHG treated women were also cured or improved, primary SUI women can be offered PAHG as a safe and durable alternative treatment.

**Abbreviations and Acronyms**
ITT: intention-to-treat
PAHG: polyacrylamide hydrogel
SUI: stress urinary incontinence
TVT: tension-free vaginal tape
VAS: visual analogue scale

Stress urinary incontinence (SUI), defined as involuntary leakage on exertion or on sneezing or coughing, is a common and distressing symptom affecting 1 of 3 adult females.^1^ Pelvic floor muscle training is the first-line treatment for SUI,^2^ and if this fails, mid urethral sling surgery with retropubic tension-free vaginal tape (TVT) has been the most common surgical option worldwide.^3^ While the efficacy of TVT is as high as 90%, the safety of mesh implants for SUI has come under scrutiny due to severe complications, and there remains uncertainty on the long-term safety profile of mid urethral slings.^3^ Thus, medical authorities in some countries have published statements and warnings about the use of mid urethral slings, and in England a national “pause” was announced for mesh slings in July 2018 that continues to date.^4,5^

Bulking agents are used for SUI treatment to create an artificial mass in the urethral submucosa and improve urethral coaptation and restore continence.^6,7^ Although the efficacy of bulking is traditionally considered to decrease over time, bulking is becoming a popular choice for patients seeking treatment for SUI.^8^ Transurethral polyacrylamide hydrogel (PAHG, Bulkamid®) is a bulking agent that has been used for more than 10 years to treat SUI in women showing high short-term subjective success rates.^9–11^ However, the long-term patient satisfaction, safety and efficacy of this treatment in primary SUI are undefined.

We report here midterm data from a randomized prospective trial comparing TVT and PAHG treatments in women with primary SUI. The aim of this study was to assess the noninferiority of PAHG compared to TVT measured by patient satisfaction (visual analogue scale [VAS] 0–100) in treatment of SUI. Secondary outcomes were subjective and objective efficacy of the treatment and complications.

## Materials and Methods

### Study Design

We conducted at Helsinki University Hospital an investigator-led, prospective, randomized, controlled, parallel-group, noninferiority trial comparing TVT and PAHG for primary SUI. The study was approved by the Helsinki University Hospital Ethics Committee (IRB no. 19/13/03/03/2015), and patients gave written informed consent prior to enrollment.

### Participants and Intervention

We recruited primary SUI patients referred from primary health care and eligible for TVT operation between September 28, 2015 and March 1, 2017, as described before.^10^

All procedures were done in an outpatient setting. As a mid urethral sling TVT (TVT-Exact®, Ethicon, Somerville, New Jersey) was used under local anesthesia (70–100 ml 0.25% prilocaine with epinephrine) as originally described.^12^ Also, PAHG injections (Bulkamid, Contura, Denmark) were performed under local anesthesia using periurethral lidocaine (10 ml) injections. Hydrogel was injected under endoscopic control at 1.5 cm from the vesicourethral junction in locations “10, 2, 5 and 7 o’clock.” Both TVT and PAHG patients were discharged after successful micturition and post-void residual <200 ml. Nine urogynecologists with strong background in SUI surgery performed TVT operations, and 2 urogynecologists did PAHG injections.

The study nurse called the patients 1 month after the procedure. As a part of the protocol, after the initial PAHG treatment we offered 1 additional PAHG injection (top up) that was carried out at the 3-month visit for patients describing persisting SUI symptoms. Followup visits performed by a doctor other than the one who had done the surgery took place at 3 months, 1 year and 3 years, and patients with persisting SUI and not satisfied with the given treatment were offered the option to choose an alternative treatment (TVT or PAHG). Patients were, however, encouraged to contact the study nurse at any time if needed.

### Outcomes

The primary noninferiority outcome was patient satisfaction with treatment ≥80 as measured by a VAS from 0 (extremely unsatisfied) to 100 (extremely satisfied) at followup visits. Secondary outcome measures were effectiveness in reducing urinary leakage and complications. We measured subjective cure with a 5-point Likert scale (symptoms cured, improved, no change, cannot tell or worsened), while a negative cough stress test with a bladder volume of 250–300 ml in a semilithotomy position and a pad stress test were used for objective cure.^13^ Complications were grouped as perioperative, early postoperative (<3 months) and later postoperative (>3 months to 3 years). We also classified complications using the Clavien-Dindo grading system.^14^ *De novo* urge was defined as the need for anticholinergic or beta-3 agonist treatment.

### Statistical Analyses

All group comparisons are presented with 2-sided 95% confidence intervals using the Newcombe method. In the study protocol calculating 1-sided confidence intervals were planned. However, during the reporting phase it was decided to use 2-sided CIs to help clinical interpretation of the results.

**Figure 1.**
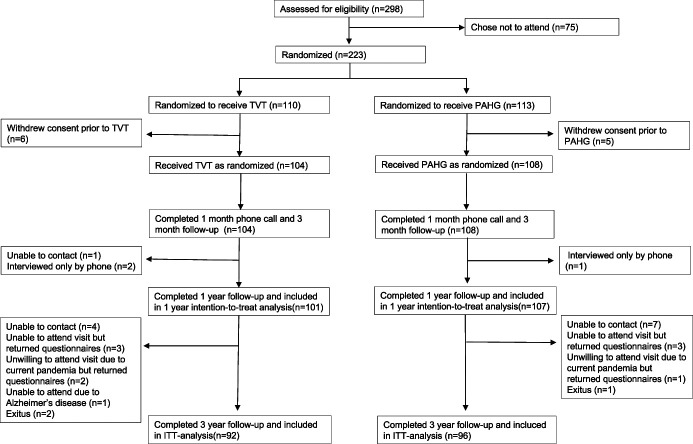
Trial profile.

As primary analysis the data are presented on an intention-to-treat (ITT) basis where the results are shown according to the assigned treatment (Fig. 1). Results were based on observed outcomes, and all patients who attended the 3-year followup visit were analyzed for results and complications (188). As a secondary analysis we analyzed primary outcome and efficacy results per protocol (treatment received) excluding women who had received other than the originally randomized treatment. Complications were calculated as before and after any crossover from the originally randomized treatment to the other, aiming to accurately relate the complication with the treatment.

All statistical reporting were calculated with SPSS® Statistics for Windows®, version 24.0 (SPSS Inc., Chicago, Illinois). There were no interim analyses planned or carried out.

### Sample Size

We estimated that patient satisfaction with the treatment result of ≥80 on a VAS from 0 to 100 was a good outcome. The sample size was calculated to test 2 portions for noninferiority, setting the patient satisfaction with the treatment 80% in the TVT group and 75% in the PAHG group based on earlier studies, ie assuming 5% unit predominance for TVT group.^15–17^ The significance level was set at 5%, power at 80% and noninferiority limit (threshold) at 20%. Altogether, 192 patients (96 per group) were required. A 10% dropout rate was assumed, and thus 212 women were planned to be randomized (1:1) for the study. Due to simultaneous recruitment by several doctors at the outpatient clinic, 223 patients were randomized before the study was closed.

## RESULTS

A total of 223 women were randomized to the study, and 212 received treatment as randomized (Fig. 1). At 3-year followup 24 women were lost, leaving 188 women (TVT 92 and PAHG 96) for the ITT analysis (Fig. 1). In total 3 women in the TVT group and 30 women in the PAHG group crossed over, leaving 155 women (TVT 89 and PAHG 66) for the per-protocol (treatment received) analysis. In the TVT group 2 women crossed over to PAHG before and 1 after the 1-year followup. In the PAHG group 16 women had received TVT before and 14 after the 1-year followup.

Baseline characteristics of the women did not differ between the groups (Table 1). At 3 years, in the TVT group 3 women (3.3%) had received PAHG after TVT, whereas no woman received a second TVT treatment. In the PAHG group 31 (32.3%) women had received only 1 PAHG injection and 35 (36.5%) women had received 2 PAHG injections, 15 (15.6%) women had received TVT after 1 PAHG injection and 15 (15.6%) women had received TVT after 2 PAHG injections.

**Table 1.** Demographics of the 212 women undergoing TVT or PAHG treatment

	TVT Group	PAHG Group
No. pts	104	108
Mean±SD yrs age (range)	50.4±10.4 (32.0–78.0)	51.5±11.0 (31.0–80.0)
Mean±SD kg/m^2^ BMI (range)	24.5±3.5 (16.1–34.9)	24.8±3.6 (18.9–34.2)
No. socioeconomical status (%):		
White- and blue-collar workers	85 (81.7)	87 (80.5)
Others	19 (18.3)	21 (19.5)
Parity/delivery:		
Mean±SD (range)	2.1±1.0 (0–5)	2.1±0.9 (0–6)
No. 0 (%)	7 (6.7)	4 (3.7)
No. vaginal delivery (%)	93 (89.4)	101 (93.5)
No. cesarean section only (%)	4 (3.8)	3 (2.8)
No. incontinence duration (%):		
>1 and <2 yrs	1 (1.0)	4 (3.7)
2–5 yrs	64 (61.5)	65 (60.2)
>5 yrs	39 (37.5)	39 (36.1)
Mean±SD VAS distress from incontinence (range)*	8.0±1.4 (4–10)	8.1±1.4 (4–10)

ITT data. *BMI*, body mass index.

*One in PAHG group missing data.

**Figure 2.**
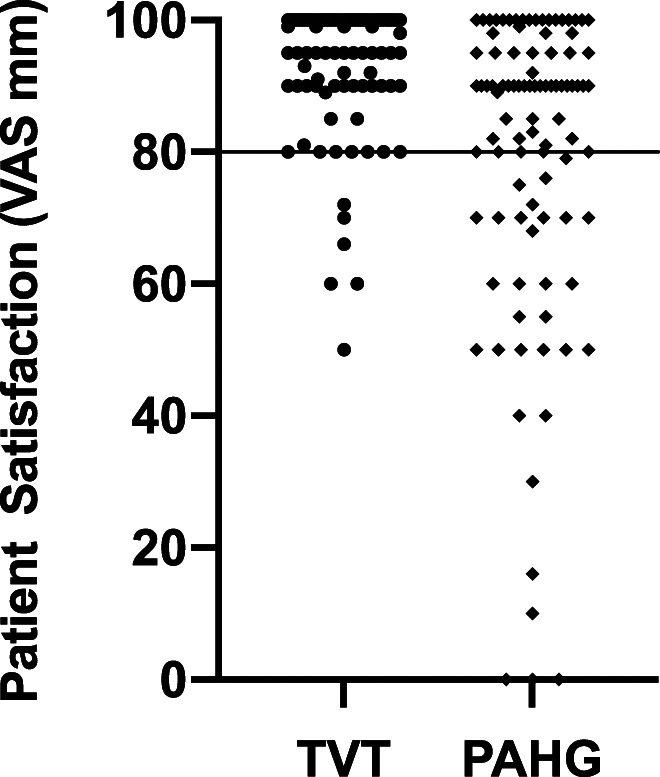
Patient satisfaction on ITT analysis 3 years after TVT or PAHG treatment.

Satisfaction median was 98.5 (IQR 90–100) in the TVT group and 90.0 (IQR 70–100) in the PAHG group at 3 years. In the TVT group 87 (94.6%) women and in the PAHG group 65 (67.7%) women scored patient satisfaction VAS ≥80 (difference 26.9%, 95% CI 16.7% to 36.8%; Fig. 2). Thus, PAHG did not meet the noninferiority criteria set in our study. More women in the TVT group than PAHG group would come again for the treatment, at 88 (95.7%) vs 78 (81.3%; difference 14.4%, 95% CI 5.9% to 23.4%) or recommend it to a friend, at 91 (98.9%) vs 81 (84.4%; difference 14.5%, 95% CI 7.4% to 23.0%). Subjective and objective cure rates are presented in Table 2.

**Table 2.** Subjective and objective cure from ITT analysis at 3 years following TVT or PAHG treatments

	No. TVT Group (%)	No. PAHG Group (%)	% Difference (95% CI)
Pts	92	96	
Subjective cure:			
Cured	55 (59.8)	24 (25.0)	34.8 (20.8–46.9)
Improved	37 (40.2)	61 (63.5)	−23.3 (−36.3–−9.0)
Cured or improved	92 (100)	85 (88.5)	11.5 (5.1–19.4)
Objective cure:			
Cough stress and pad test neg	76 (82.6)	47 (50.0)	33.7 (20.3–45.3)
Cough stress test neg	88 (95.7)	75 (78.1)	17.5 (8.0–27.2)
Pad test neg	76 (83.5)	53 (56.4)	27.4 (14.3–39.2)

In per-protocol (treatment received) analysis satisfaction median was 99.0 (IQR 90–100) in the TVT group and 85.0 (IQR 69.5–90) in the PAHG group, and 89 (100%) and 56 (84.8%; difference 15.2%, 95% CI 7.7% to 25.7%) considered themselves subjectively cured or improved, respectively. Objective cure measured by negative cough stress and pad test was reached in 74 (83.1%) women in the TVT group and 22 (33.3%) women in the PAHG group (difference 49.8%, 95% CI 34.7% to 61.8%).

Within the 3-year followup period any peri- or postoperative complication was detected in 40 (43.5%) women in the TVT group and 23 (24.0%) women in the PAHG group before crossover (difference 19.5%, 95% CI 6.8% to 31.4%). Complications were detected in the TVT group vs PAHG group perioperatively in 18 (17.3%) vs 2 (1.9%) women, <3 months postoperatively in 13 (12.5%) vs 9 (8.3%) women and >3 months to 3 years 20 (21.7%) vs 14 (14.6%) women, respectively (Table 3). Two or more complications occurred in 10 (10.9%) women in the TVT group and 2 (2.1%) women in the PAHG group (difference 8.8%, 95% CI 1.7% to 16.9%).

**Table 3.** Complications before crossover

	No. TVT Group (%)	No. PAHG Group (%)	% Difference (95% CI)
Pts	104	108	
Periop complications:			
Hematoma	6 (5.8)	0	5.8 (1.1–12.0)
Bladder perforation	7 (6.7)	0	6.7 (1.9–13.2)
Acute urinary retention	10 (9.6)	2 (1.9)	7.8 (1.4–15.1)
Complications occurring <3 mos postop:			
Vaginal tape extrusion	3 (2.9)	0	2.9 (−1.0–8.1)
Reop due to hematoma	1 (1.0)	0	1.0 (−2.6–5.2)
Reop due to retention	3 (2.9)	0	2.9 (−1.0–8.1)
Urinary tract infections	7 (6.7)	9 (8.3)	−1.6 (−9.2–6.0)
Complications occurring >3 mos–3 yrs postop:*			
Vaginal tape erosion	3 (3.3)	0	3.3 (−1.1–9.2)
Reop due to erosion	3 (3.3)	0	3.3 (−1.1–9.2)
Reop due to pain	0	1 (1.0)	−1.0 (−5.7–3.1)
Reop due to fibrotic resistance	0	1 (1.0)	−1.0 (−5.7–3.1)
Tape/implantation site pain	6 (6.5)	1 (1.0)	5.5 (−0.3–12.5)
Dysuria	4 (4.3)	1 (1.0)	3.3 (−2.0–9.7)
Post-void residual >150 ml	1 (1.1)	0	1.1 (−2.9–5.9)
*De novo* urgency	9 (9.8)	12 (12.5)	−2.7 (−12.0–6.6)
Clavien-Dindo distribution of complications at 3 yrs:*			
None	61 (66.3)	78 (81.3)	−14.9 (−27.0–−2.4)
Grade I	11 (12.0)	1 (1.0)	10.9 (4.0–19.2)
Grade II	13 (14.1)	15 (15.6)	−1.5 (−11.8–8.9)
Grade III†	6 (6.5)	2 (2.1)	4.4 (−1.8–11.6)
Grade IV‡	1 (1.1)	0	1.1 (−2.9–5.9)

*Ninety-two patients in TVT group, 96 in PAHG group.

† Acute urinary retention with loosening and ultimately cutting the tape (1 patient), blood transfusion and laparotomy due to hematoma (1), acute urinary retention with loosening the tape using local anesthesia (2), repair of tape extrusion using local anesthesia repeatedly (1), repair of tape erosion (1), after PAHG attempt to aspirate fibrotic resistance at bladder neck using local anesthesia (1), removal of PAHG due to persisting pain in general anesthesia (1).

‡ Ischemic heart symptoms postoperatively possibly related to local anesthesia.

Before any crossover, 6 (6.5%) women in the TVT group and 1 woman (1.0%) in the PAHG group (difference 5.5%, 95% CI −0.3% to 12.5%) had reported pain at the tape/implantation site within the 3-year followup period. The numerical rating scale (0–10) median for the patients reporting pain at 3 years was 3 (IQR 2–4). In the TVT group 4 women (4.3%) and in the PAHG group 1 woman (1.0%) reported dysuria before crossover (difference 3.3%, 95% CI −0.2% to 9.7%). One woman requested removal of PAHG due to dysuria and implantation site pain.

After crossover from TVT to PAHG (3), 1 woman had postoperative retention. After crossover from PAHG to TVT (30), 12 women had complications at 3-year followup, including 2 reoperations (hematoma and loosening of tape), 1 erosion, 1 tape site pain, 3 women with dysuria, 2 women with retention and 3 women with *de novo* urgency.

The Clavien-Dindo classification showed significantly more grade I complications with TVT (11, 12.0%) compared to PAHG (1, 1.0%; difference 10.9%, 95% CI 4.0% to 19.2%), but not in the more severe complications (Table 3). However, complications requiring reoperations occurred in 7 women after TVT surgery, whereas 2 PAHG patients had a complication-related reoperation.

## DISCUSSION

In a randomized clinical trial, we show that at the 3-year followup VAS satisfaction median in both treatment groups was high, being 99 in the TVT group and 90 in the PAHG group. However, at the primary endpoint in our study, satisfaction VAS ≥80 was reached in 95% of the TVT group compared with 68% in the PAHG group, and thus TVT achieved a better outcome. On the other hand, complications were more often associated with TVT than with PAHG.

The long-term durability of the TVT-procedure has been documented consistently,^18–20^ and our current data further support this. Recently, concern has risen about potential long-term complications with mid urethral slings, such as pain, dysuria and mesh erosions.^21,22^ This has also affected new recommendations, eg NICE (National Institute for Health and Care Excellence) guidelines advise that mid urethral slings should only be considered if alternative surgical procedures are not suitable.^23^ Data from routine practice indicate that the risk of mid urethral sling removal for any reason was 2.7% at 5 years and 3.3% at 9 years.^24^ Another study demonstrated a 2.9% rate of long-term sling-specific surgical intervention.^19^ In our study, tape extrusion/impaired healing occurred in 2.9% of women and erosion requiring reoperation in 3.3% of women, but none of the women had the sling removed.

The number of complications in our study was low with both treatments. Since crossover in the PAHG group was more common than in the TVT group, our focus was on complications after the initial treatment in order to accurately relate the complication with the treatment. Major complications such as reoperations were mainly associated with TVT. Furthermore, the difference in complications between the treatments is also shown by our finding that patients having more than 1 perioperative or postoperative complication were detected more likely after TVT (10) than PAHG (2). The typical TVT-related complications, such as pain, dysuria, and mesh erosions, were also seen in our data, with similar rates as in previous studies.^25^

Pain has become one of the most feared complications after mesh surgery.^25^ In our study, within the 3-year followup, 6 patients reported pain after TVT, but none of these patients requested tape removal. One PAHG patient reported pelvic pain and dysuria 20 months after the primary injection and requested PAHG removal that did not affect the pain. She had a history of pelvic pain after delivery, and thus explicit association of the pain and dysuria to PAHG remains uncertain. The other PAHG-related complication requiring intervention (aspiration using local anesthesia) was a vaginal fibrotic resistance below the urethra in an asymptomatic woman. Although the exact etiology is unknown, it could be a PAHG injection-related reaction due to, for example, hematoma and/or local infection. It should be noted that several previously used bulking agents have been associated with more severe complications resulting in the withdrawal of these treatments from the market.^26^ Our study supports the existing data that with PAHG, complications are mostly mild and/or transient.^26^

*De novo* urgency, defined as the need for medication, did not differ between the groups and occurred at a similar rate as seen in previous studies with TVT.^3,20^ The finding that *de novo* urgency was detected after PAHG in a similar manner supports the general mechanism of obstruction for *de novo* urgency. Since urodynamics were not routinely performed, underlying urgency cannot be ruled out.^27^

Some previous studies with short-term followup have suggested that the efficacy of PAHG in SUI treatment might be transient, thus resulting in inadequate cure and subjective satisfaction.^28^ In a 12-month randomized clinical trial where PAHG and collagen gel were compared, 77% of the women with PAHG considered themselves cured or improved, despite the fact that only 24% were objectively cured.^9^ In our trial we detected a similar pattern as in the North American study^9^ since although only 54% of the women were objectively cured, 92% considered themselves cured or improved.^10^ Importantly, after 3 years this high satisfaction rate was sustained with PAHG, as 89% of the women still considered themselves cured or improved compared to 92% at 1 year, while 50% were objectively cured compared to 54% at 1 year. Thus, in SUI treatment high subjective satisfaction does not seem to always require complete objective cure. When women were asked about attitudes and expectations with different treatments, 57% preferred a minor procedure with low risk of complications, and they were prepared to accept a lower success rate.^29^ Thus, health care providers should share both objective and subjective data of the different surgical interventions in order for women to make informed decisions based on their personal goals and expectations.

The main strengths of our trial are comparison of the gold standard surgical SUI treatment with a minimally invasive SUI treatment, and the midterm followup with both subjective and objective outcomes. Furthermore, our recruited participants represent a nonselected population of women considering surgical treatment for primary SUI. In our study, the dropout and loss to followup rates were low. Thus, we provide robust data to inform both health care providers and women considering invasive SUI treatment.

Our study also has limitations. Since our patients were primary SUI patients, we did not perform invasive urodynamics in all women. This is also our normal clinical practice as most guidelines follow the strong evidence that invasive urodynamics do not provide any further benefit after detailed office evaluation in primary SUI patients.^30^ Neither did we segregate women based on urethral hypermobility or intrinsic sphincter deficiency since each is often present to some extent in typical SUI patients.^6^ Furthermore, we did not include women with clinically significant pelvic organ prolapse or obese women since concomitant prolapse surgery as well as severe obesity could potentially affect the SUI treatment results. Our study was unblinded to the treatment; however, blinding is practically impossible as the treatments were performed with local anesthesia. Although our data cannot be fully generalized to all other mid urethral slings or bulking agents, we studied TVT, which is the gold standard for SUI, and PAHG, being currently the most commonly used bulking agent in Europe. And finally, the study was carried out in a university hospital with urogynecologists who have a strong experience in surgical SUI treatment. This may partially explain our high success rates with both treatments that may not be fully comparable to results at smaller clinical units. However, we believe that with adequate training and similar procedure volumes, comparable results are achievable.

## CONCLUSIONS

In this randomized clinical trial with midterm followup, PAHG did not reach the noninferiority criteria in patient satisfaction. However, most PAHG-treated women considered themselves cured or improved, and most importantly, the vast majority of women were satisfied without the need for further invasive treatment. Although overall complication rates were low in our trial, severe complications were almost exclusively associated with TVT. These findings will help women make informed decisions based on their personal goals and expectations when considering invasive SUI treatment.
